# Intrauterine Device Insertion during Cesarean Section in Women without Prenatal Contraception Counseling: Lessons from a Country with High Cesarean Rates

**DOI:** 10.1055/s-0039-1693677

**Published:** 2019-08

**Authors:** Alberto Moreno Zaconeta, Ana Carolina Oliveira, Flavielly Souza Estrela, Thalia Maia Vasconcelos, Paulo Sergio França, Miriam da Silva Wanderley, Angelica Amorim Amato

**Affiliations:** 1 Area of Gynecology and Obstetrics, Medical School, Universidade de Brasília, Brasília, DF, Brazil; 2 Laboratory of Molecular Pharmacology, Faculdade de Ciências da Saúde da Universidade de Brasília, Brasília, DF, Brazil

**Keywords:** intrauterine device, cesarean section, postplacental intrauterine device insertion, prenatal contraception counseling, intrauterine device continuation, dispositivo intrauterino, cesárea, inserção de dispositivo intrauterino pós-dequitação, aconselhamento contraceptivo pré-natal, continuidade do dispositivo intrauterino

## Abstract

**Objective** The moment of admission for delivery may be inappropriate for offering an intrauterine device (IUD) to women without prenatal contraception counseling. However, in countries with high cesarean rates and deficient prenatal contraception counseling, this strategy may reduce unexpected pregnancies and repeated cesarean sections.

**Methods** This was a prospective cohort study involving 100 women without prenatal contraception counseling. Postplacental IUD was offered after admission for delivery and placed during cesarean. The rates of IUD continuation, uterine perforation, and endometritis were assessed at 6 weeks and 6 months, and the proportion of women continuing with IUD at 6 months was assessed with respect to the number of previous cesareans.

**Results** Ninety-seven women completed the follow-up. The rate of IUD continuation was 91% at 6 weeks and 83.5% at 6 months. The expulsion/removal rate in the first 6 weeks was not different from that between 6 weeks and 6 months (9 vs 9.1%, respectively). There were 2 cases of endometritis (2.1%), and no case of uterine perforation. Among 81 women continuing with intrauterine device after 6-months, 31% had undergone only the cesarean section in which the IUD was inserted, 44% had undergone 2 and 25% had undergone 3 or more cesarean sections.

**Conclusion** Two thirds of the women who continued with IUD at 6 months had undergone 2 or more cesarean sections. Since offering trial of labor is unusual after 2 or more previous cesareans, we believe that offering IUD after admission for delivery may reduce the risk of repeated cesarean sections and its inherent risks.

## Abstract (pt)

**Resumo:**

**Objetivo** O momento da admissão para o parto pode ser considerado inapropriado para oferecer o dispositivo intrauterino (DIU) para mulheres sem aconselhamento contraceptivo pré-natal. Entretanto, em países com elevadas taxas de cesáreas e aconselhamento contraceptivo deficiente, essa estratégia pode reduzir o risco de gestações não programadas e cesáreas repetidas.

**Métodos** Estudo de coorte envolvendo 100 mulheres sem aconselhamento contraceptivo pré-natal. A inserção de DIU pós-dequitação foi oferecida após a admissão para o parto e indicação de cesárea. As taxas de continuidade com o DIU, perfuração uterina e endometrite foram avaliadas após 6 semanas e 6 meses, e a proporção de mulheres que continuaram com o DIU após 6 meses foi analisada em relação ao número de cesáreas prévias.

**Resultados** Noventa e sete mulheres completaram o seguimento. A taxa de permanência do DIU foi de 91% em 6 semanas e 83,5% em 6 meses. A taxa de expulsão/remoção nas primeiras 6 semanas foi não foi diferente daquela observada entre 6 emanas e 6 meses (9 vs 9,1%, respectivamente). Houve dois casos de endometrite (2,1%), e nenhum caso de perfuração uterina. Entre as 81 mulheres que permaneceram com o DIU após 6 meses, 31% haviam sido submetidas a apenas uma cesárea, em que o DIU foi inserido, 44% a 2, e 25% a 3 ou mais cesáreas.

**Conclusão** Dois terços das mulheres que continuaram com o DIU após 6 meses haviam sido submetidas a 2 ou mais cesáreas. Considerando que oferecer a tentativa de parto vaginal após duas ou mais cesáreas prévias é incomum, é possível que a oferta do DIU na admissão para o parto possa reduzir o risco de cesáreas repetidas e de seus riscos associados.

## Introduction

Intrauterine device (IUD) is an effective contraceptive method for postpartum period, with the advantages over hormonal methods of being independent of women's compliance and not affecting the coagulation system or lactation.^1^

Intrauterine device placement is usually performed 6 weeks following delivery (interval insertion), due to evidence indicating a lower expulsion rate when compared with immediate postplacental insertion.^2^ However, in real life setting, women experience difficulties to return for a postpartum visit, and it was reported that almost half of the women who had the intention of using IUD for postpartum contraception turned out not to have an IUD inserted.^3^ In face of these limitations of interval IUD placement, there has been growing interest on immediate insertion. Immediate insertion is associated with an overall low expulsion rate, of around 10%,^4^ which is significantly lower when following cesarean when compared with vaginal delivery.^4^ ^5^ ^6^

Brazil has an estimated population of over 200 million inhabitants and is among the countries with the highest cesarean delivery rate.^7^ Despite most women having access to prenatal care in Brazil,^8^ postpartum contraception is not discussed frequently,^9^ and health care providers view the moment of delivery as an inadequate setting to provide information about IUD and for women to decide whether they want it to be inserted or not.

In Brazil, almost two thirds of the women admitted to delivery are aged under 29 years old.^10^ Moreover, cesarean delivery rates are around 56%, and there is an established practice of avoiding vaginal delivery in women with 2 or more previous cesarean sections. It is, therefore, possible that offering postplacental IUD insertion to women who are going to have a cesarean delivery, regardless of whether or not they received prenatal contraception counseling, would be an effective strategy to avoid repeated cesarean sections in young women.

The primary objective of this study was to determine the rates of IUD continuation, uterine perforation and endometritis after 6 weeks and 6 months in women for whom IUD contraception was offered at admission for delivery, immediately after the indication of a cesarean section, and inserted after placental delivery. The secondary objective was to estimate the proportion of women for whom the above mentioned strategy of immediate IUD insertion potentially avoided a third or fourth cesarean section.

## Methods

This was a prospective cohort study conducted between February 2012 and June 2013, at the Hospital Universitário de Brasília, DF, Brazil. It was approved by the Ethics Committee (register number 183/11) of the institution and conducted according to the Strengthening the Reporting of Observational Studies in Epidemiology (STROBE) guidelines for cohort studies. Pregnant women aged 18 years or over for whom cesarean delivery was indicated after admission were included when both the woman and the medical staff on duty agreed to participate. The exclusion criteria were as follows: women with a personal history of dysmenorrhea or menorrhagia, with a high risk of sexually transmitted disease (arbitrarily defined as more than one sex partner over the last 6 months or over the period of 6 months preceding pregnancy, or a positive human immunodeficiency virus, venereal disease research laboratory or hepatitis B virus antigen test during pregnancy), gestational age of less than 32 weeks at the time of delivery, fever over the last 48 hours, membrane rupture for over 12 hours before delivery and signs of vaginitis or cervicitis on gynecological examination.

Immediately after indication of cesarean section, the women were offered the possibility of IUD insertion during surgery, and all their doubts about this contraception method were clarified. Those who agreed with insertion signed a written informed consent. Following placental delivery, the IUD (model TCu 380A, Injeflex, São Paulo, SP, Brazil) was inserted using a standard technique: while one of the surgeon's hand held the outer face of the fundus of the uterus, the other hand, with the IUD between the second and third fingers, inserted the device in the fundus through the hysterotomy incision and, after this, directed the strings to the cervix, without cutting them. The IUD was inserted by the medical residents or staff on duty, who had received a brief training provided by the researchers. All participants received one to two grams of cefazolin prophylaxis before cesarean section.

To avoid follow-up losses, all participants received a phone number to contact the researchers over the following 6 months, if necessary. The women returned for postpartum visits with the researchers after 6 weeks and 6 months and underwent gynecological examination and transvaginal sonography at the Hospital Universitário de Brasília. For women who had not undergone oncologic colpocytology assessment during pregnancy, this was done in the 6-week visit. When the participant missed one of the postpartum visits and phone contact was unsuccessful, the researchers visited the women in her residency to make the visit to the hospital feasible.

The IUD was considered adequately positioned at the 6-week and 6-month visits if it was inside the uterine cavity, above the internal cervical os. Expulsion was defined as total exteriorization of the IUD or when transvaginal sonography showed the device was inside the cervical canal. In the latter situation, the IUD was immediately extracted. Intrauterine device removal was defined by removal of the device, due to any reason, when it was situated above the internal cervical orifice. Statistical analysis was conducted using Graphpad Prism software, version 7.0 (GraphPad Software Inc., La Jolla, CA, USA). The Chi-squared and Fisher exact tests were used to compare proportions, and one-way analysis of variance (ANOVA) was used to compare means. Statistical significance was considered when the *p*-value was lower than 0.05.

## Results

One hundred women were included in the study, and their characteristics are presented in table 1. The mean age of the participants was 27.7 (±5.6) years (95% confidence interval 26.6–28.7 years). Two thirds of the participants had at least a previous cesarean section, and one in every five participants had two or more previous cesarean sections (Table 1).

**Table 1:** Characteristics of the women included in the study (n = 100)

Variables	N (%)
Age (years)	
< 25	33 (33)
25-34	52 (52)
≥ 35	15 (15)
Previous deliveries	
None	21 (21)
1 or more	79 (79)
Previous cesarean sections	
None	33 (33)
1	45 (45)
2 or more	22 (22)
Gestational age at delivery (weeks)	
< 37	5 (5)
37-40 + 6	77 (77)
41 or more	18 (18)

**Fig. 1:**
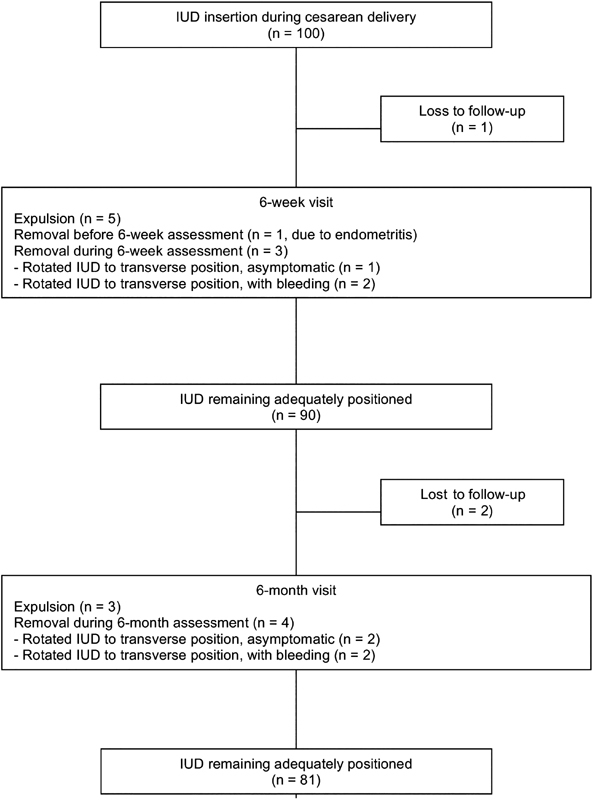
Six-month follow-up of 100 participants for whom IUD insertion was offered immediately after the indication of a cesarean section and inserted after placental delivery.

Among the 100 participants, 99 returned for the 6-week visit (Fig. 1). Five of them (5.1%) presented IUD expulsion, and one underwent IUD removal due to endometritis. Two women requested IUD removal at this visit due to excessive bleeding, and one requested removal after being informed that the device was rotated to the transversal position (i.e. the main axis of the IUD was perpendicular to the main uterine axis), despite being asymptomatic. Therefore, 90 (90.9%) women remained with the IUD after 6 weeks of insertion during cesarean delivery (Fig. 1).

A total of 88 women returned for the 6-month visit, and 3 (3.4%) of them had IUD expulsion. One woman presented endometritis between the 6-week and 6-month visits and was treated with antibiotics without IUD removal, with good response. Two women requested IUD removal at this visit because of excessive bleeding, and two requested removal after being informed that the device was rotated to the transversal position, despite being asymptomatic. Therefore, 81 (83.5%) of the participants remained with the IUD after 6 months (Fig. 1). Among these women, the strings were visible during gynecological assessment in 31 (38.3%).

The expulsion/removal rate during the first 6 weeks after insertion (9.1%) was similar to that observed between 6 weeks and 6 months of insertion (9%, *p* > 0.05). When only women presenting expulsion were considered, there was also no difference between both time points (5.1% at the 6-week visit vs. 3.4% at the 6-month visit, *p* > 0.05). Among the 97 women who completed follow-up, 81 (83.5%) remained with the IUD 6 months after cesarean delivery, 8 (8.2%) presented expulsion, and 8 (8.2%) requested its removal. There were no cases of uterine perforation. There was no difference between women who remained with the IUD at 6 months and women with IUD expulsion/removal with respect to age, parity, the number of previous cesarean deliveries or gestational age at delivery (Table 2).

**Table 2:** Comparision between the women who remained with de intrauterine device after a six-month follow-up and those who presented expulsion/removal

Variables	With IUD (n = 81)	Without IUD (n = 16)	*P*-value
Age (mean ± SD)	29.1 ± 6.3	27.5 ± 5.5	*p* = 0.322^a^
Age (years)			
< 25	27 (32.9%)	5 (33.3%)	*p* = 0.3187^b^
25–34	43 (52.4%)	7 (46.7%)	
≥ 35	12 (14.6%)	3 (20.0%)	
Previous deliveries			
None	16 (19.5%)	5 (33.3%)	*p* = 0.3046^c^
1 or more	66 (80.5%)	10 (66.7%)	
Previous cesarean sections			
None	25 (30.5%)	7 (46.7%)	*p* = 0.2193^b^
1	36 (43.9%)	7 (46.7%)	
2 or more	21 (25.6%)	1 (6.6%)	
Gestational age at delivery (weeks)			
< 37	4 (4.9%)	1 (6.7%)	*p* = 0.8723^b^
37–40 + 6	63 (76.8%)	12 (80%)	
41 or more	15 (18.3%)	2 (13.4%)	

Abbreviations: IUD, intrauterine device, SD, standard deviation.

a Student *t*-test;

b Chi-square test;

c Fisher exact test.

**Fig. 2:**
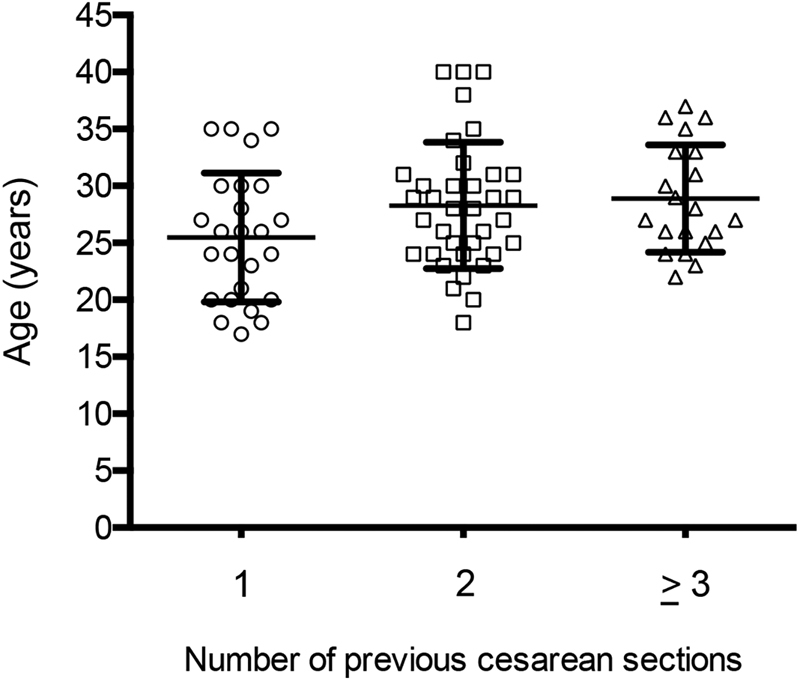
Age distribution of women with one, two, or three or more cesarean sections. The mean ± SD was 25.4 (±5.6), 28.2 (±5.5) and 28.9 (±4.7) years, respectively (*p* > 0.05 by one-way analysis of variance).

Among the 81 women who remained with the IUD 6 months following the delivery, 25 (30.8%) had undergone only the cesarean section in which the device was inserted, 36 (44.4%) had undergone 2 cesarean sections, 18 (22.2%) had undergone 3 cesarean sections, and 2 (2.5%) had undergone 4 cesarean sections. Therefore, taking into account that offering vaginal delivery for women who have undergone 2 or more previous cesarean sections is exceptional in Brazil, IUD insertion hypothetically prevented the 3^rd^, 4^th^ and 5^th^ cesarean sections in 36, 18 and 2 of the participants, respectively. There was no statistically significant difference among women who had undergone one, two, three or more cesarean sections with respect to age (Fig. 2).

**Fig. 3:**
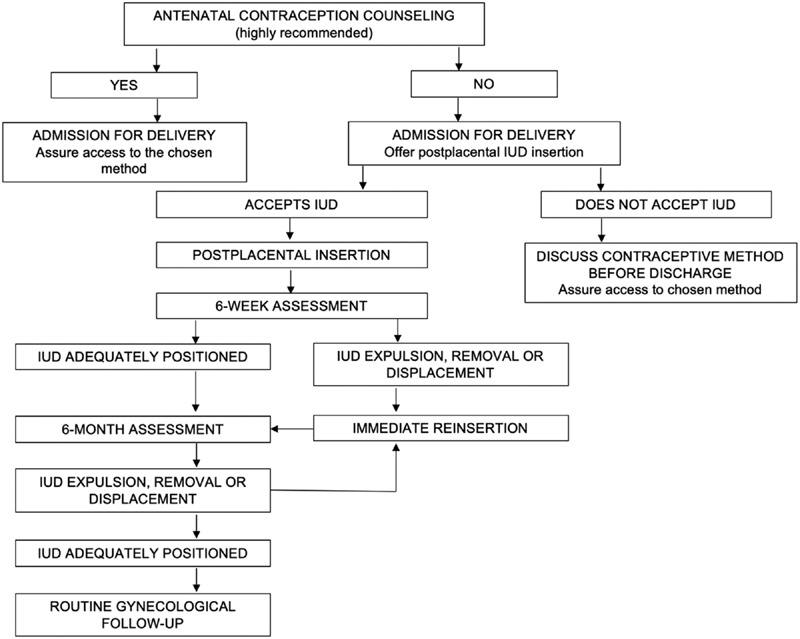
Suggested approach for offering postpartum contraception. IUD: Intrauterine device.

## Discussion

In the present study, we found that offering IUD contraception immediately before cesarean delivery, followed by device insertion during surgery, was an effective strategy, since after 6 weeks the rate of IUD permanence was 90%. This strategy is also possibly more convenient, because women who wish to use IUD contraception do not need to return early in the postpartum, a period with well-known difficulties.^3^ Moreover, the fact that there was no difference in the expulsion rate during the first 6 weeks and in the period between 6 weeks and 6 months reinforces the convenience of IUD insertion during surgery, when the uterus is literally “in the hand and open”, and the procedure can be carried out without any additional expense.

Three previous studies, 2 conducted in Brazil and the other in North America, reported a 100% rate of IUD continuation 6 months after insertion during cesarean section.^6^ ^11^ ^12^ However, 2 studies assessed a limited number of women (19 and 25),^6^ ^11^ and in the other one, 52% of the 90 women included were lost to follow-up.^12^ Since the rate of IUD expulsion is overall low, it is expected that small samples comprise more frequently women in whom the IUD remained in the uterus. In addition, large losses to follow-up may impair data interpretation, because it is not possible to conclude that the women who did not return for follow-up were those more often presenting IUD expulsion. These aspects may explain the differences between the findings from previous studies^6^ ^11^ ^12^ and our data. We assessed 100 women and had a low rate of loss to follow-up (3%). Intrauterine device permanence rates after 6 weeks (90%) and 6 months (81%) were similar to those reported by Çelen et al (93% and 82%, respectively), who assessed 245 women and had no losses to follow-up.^13^

Levi et al^14^ recently reported an 83% IUD continuation rate after 6 months among women randomly assigned for IUD insertion during cesarean section. Despite the similarity with our finding, the studies have methodological differences. In particular, Levi et al^14^ inserted another IUD in the 3 participants presenting expulsion and in the participant from whom the initial device was removed due to endometritis, and these 4 women were considered as cases of IUD permanence at 6 months. In the current study, we reported the continuation rate of IUD exclusively inserted during the cesarean section, and it could be expected that if the expulsed or removed IUDs had been replaced the rate at 6 months would have been higher. It is important to point that from a public health perspective, immediate IUD replacement after expulsion or removal could be an interesting strategy for increasing the continuation rate in women who had the IUD inserted during cesarean delivery.

Another approach to increase the continuation rate would be to avoid unnecessary removal of IUDs inserted during cesarean sections. Two participants (2.1%) included in our study presented endometritis, one of them underwent IUD removal, and the other one maintained the IUD and showed a good response to antibiotic treatment. There are currently no data to provide definitive recommendations for or against IUD maintenance in women with endometritis, and, therefore, the best approach for treating these women is based on clinical judgement. The Center for Diseases Control and Prevention from North America recommends that women outside the puerperium with inflammatory pelvic disease receiving IUD contraception should be treated with antibiotics, and the device should be removed only if there is no satisfactory response to therapy after 48to 72h.^15^ We believe that this recommendation could be extended to women with endometritis following cesarean delivery, since this approach would enable the assistant physician to determine, on an individual basis, those women for whom antibiotic therapy should be best accompanied by IUD removal.

An unexpected finding in our study was that eight women exhibited IUD rotation to a transversal situation inside the endometrial cavity in sonographic assessment. According to criteria established in the study design, they were considered to be well positioned, but, on the basis of radiologic criteria, rotated devices are considered malpositioned due to displacement.^16^ This finding was informed to all eight participants, and three of them decided to remove the IUD despite being asymptomatic. To our knowledge, this situation was not reported in previous studies. Outside the puerperium, IUD rotation is associated with pain and bleeding,^16^ and, in the current study, two participants exhibiting IUD rotation requested its removal due to bleeding. We, therefore, suggest that women showing IUD rotation and presenting bleeding or feeling unsafe should undergo prompt IUD replacement.

We did not observe any case of uterine perforation, a complication that was also not reported in a study involving over 17,000 women undergoing IUD insertion during cesarean section in 6 different countries.^5^

Six months after insertion, IUD strings were visible in 38% of the participants with well-positioned device. In this same time period and using the same IUD model, data from other studies indicated visible strings in between 40^14^ and 78% of the participants.^17^ Both women and health care providers should acknowledge that the strings may not be visible in appropriately positioned IUDs that were inserted during cesarean delivery.

By offering IUD insertion to women who had received no prenatal contraception counseling, we challenged a common dogma in Brazil: that the time point immediately before cesarean delivery is an inadequate moment for the women to receive counseling and freely decide about IUD insertion. Data from Brazilian studies indicate that most women do not receive prenatal contraception counseling.^9^ Among those who do, after delivery, only one third has access to the contraceptive method that was chosen, with contraceptive injection being the method showing higher concordance between the women's previous choice and the method used in the puerperium, and IUD showing the lowest concordance in this respect.^18^ Although we believe that contraception counseling should be routinely provided in prenatal care because this is the most effective postpartum contraception strategy,^5^ ^19^ we acknowledge that not offering IUD insertion at the moment of delivery only because the method was not discussed during the pregnancy represents, indeed, a “second failure” in providing appropriate contraception counseling.

The current study sought solutions for a common problem in public Brazilian maternities. Since Brazil is the second country with the higher cesarean section rate worldwide,^7^ with a population of young parturients, and a law system that restricts surgical sterilization during cesarean delivery, it is common that pregnant women younger than 30 years old have a previous history of one or more cesarean deliveries. Additionally, because vaginal delivery is exceptionally offered for women who have undergone two or more cesarean sections, thousands of young women are annually exposed to the risks of repeated cesarean sections. In the current study, we strongly believe that the cases in which the IUDs remained in the endometrial cavity after 6 months of insertion potentially prevented an additional cesarean section in two thirds or the participants, since 68% of women were undergoing the 2^nd^, 3^rd^ or even 4^th^ cesarean delivery at the moment of IUD insertion.

Women undergoing repeated cesarean sections are at increased risk of bowel and urinary tract injury, but the most feared complication is placenta accreta,^20^ which is associated with high morbidity and mortality. Due to the dramatic increase in the frequency of placenta accreta in the United States over the last two decades, the implementation of centers of excellence for managing this condition was recently proposed.^21^ Despite the reduction in morbidity and mortality when women with established placental accretism are managed in these high complexity centers (tertiary prevention), providing effective contraception would be an effective mean to promote primary prevention of this condition.

It is important to point that, in the present study, there was a great effort to avoid losses to follow-up, particularly at the 6-week visit. This is a time period when women face difficulties in returning to the health service. Assessment at this time may, therefore, not reflect the real-life setting, and therefore reinforces the importance of assuring that the contraceptive method desired by the woman is provided at discharge.

Our findings are limited by the fact that the participants were recruited from only one center. However, the high rates of cesarean deliveries are evenly distributed in the country, and one may, therefore, speculate that the results of offering IUD insertion at the moment of the indication of cesarean section would be similar among Brazilian public maternities.

In light of our findings, we strongly believe that health care providers should not view the lack of antenatal contraception counseling as a barrier to offering IUD insertion at the time point of cesarean section indication. This approach not only enables women to enjoy their sexual and reproductive rights but potentially discontinues a myriad of health injuries. We hence propose a strategy to optimize contraception in the postpartum period in Figure 3.

## Conclusion

Our findings indicate a high rate of IUD continuation and low rates of complications after postplacental insertion for women undergoing cesarean sections in a setting of high cesarean delivery rates and deficient prenatal contraception counseling. Since offering trial of labor is unusual after two or more previous cesareans, it is possible that offering IUD after admission for delivery may reduce the risk of repeated cesarean sections and its inherent risks.
